# The Combination of *In vivo* ^124^I-PET and CT Small Animal Imaging for Evaluation of Thyroid Physiology and Dosimetry

**DOI:** 10.3390/diagnostics2020010

**Published:** 2012-06-05

**Authors:** Henrik H. El-Ali, Martin Eckerwall, Dorthe Skovgaard, Erik Larsson, Sven-Erik Strand, Andreas Kjaer

**Affiliations:** 1 Cluster for Molecular Imaging, Faculty of Health Sciences, University of Copenhagen, Copenhagen DK-2200, Denmark; E-Mails: dskovgaard@hotmail.com (D.S.); akjaer@sund.ku.dk (A.K.); 2 Department of Clinical Physiology, Nuclear Medicine & PET, Rigshospitalet, Blegdamsvej 9, Copenhagen 2100, Denmark; 3 Medical Radiation Physics, Department of Clinical Sciences, University of Lund, Lund SE-221 85, Sweden; E-Mails: martin.eckerwall@siemens.com (M.E.); erik.larsoon@med.lu.se (E.L.); sven-erik.strand@med.lu.se (S.-E.S.)

**Keywords:** thyroid rat model, rat S-values, ^124^I-thyroid imaging, molecular imaging, animal PET/CT co-registration, small animal dosimetry

## Abstract

***Objective:***A thyroid rat model combining functional and anatomical information would be of great benefit for better modeling of thyroid physiology and for absorbed dose calculations. Our aim was to show that ^124^I-PET and CT small animal imaging are useful as a combined model for studying thyroid physiology and dose calculation. ***Methods*:** Seven rats were subjects for multiple thyroid ^124^I-imaging and CT-scans. S-values [mGy/MBqs] for different thyroid sizes were simulated. A phantom with spheres was designed for validation of performances of the small animal PET and CT imaging systems. ***Results:***Small animal image-based measurements of the activity amount and the volumes of the spheres with *a priori* known volumes showed a good agreement with their corresponding actual volumes. The CT scans of the rats showed thyroid volumes from 34–70 mL. ***Conclusions:***The wide span in volumes of thyroid glands indicates the importance of using an accurate volume-measuring technique such as the small animal CT. The small animal PET system was on the other hand able to accurately estimate the activity concentration in the thyroid volumes. We conclude that the combination of the PET and CT image information is essential for quantitative thyroid imaging and accurate thyroid absorbed dose calculation.

## 1. Introduction

The aim of this work was to demonstrate the possibility and usefulness of using laboratory rats using a combination of the PET (Positron Emission Tomography) and the CT (Computed Tomography) techniques [1] as an *in vivo* animal model for the studies of thyroid physiology and dosimetry. Such a realistic, *i.e.*, translational, and applicable animal model, for real animal morphology, is essential for reliable modeling of human thyroid physiology compared with rigid thyroid phantoms. The co-registration of the ^124^I-PET and the CT modalities has previously been applied in patient studies. Since PET imaging is a translational imaging modality, the results from preclinical studies that accurately model human normal/abnormal physiological processes more reliably, can be essential for clinical applications. An *in vivo* small animal model can therefore be of great interest for a better understanding of human thyroid physiology and dosimetry. To our knowledge, no animal model combining small animal ^124^I-PET imaging and small animal CT thyroid imaging and dosimetry evaluation has been reported. The animal model was extended for individual absorbed dose calculations of the animal thyroid. The importance of small animal CT for the volume determination of the thyroid and the absorbed dose calculations was also investigated. 

Several iodine radionuclides are used for thyroid studies and treatment due to the high accumulation in the thyroid gland. Among others, ^123^I and ^131^I frequently have been used for planar imaging as well as Single Photon Emission Computed Tomography (SPECT) studies. ^123^I is a good imaging radionuclide with a low absorbed dose, although its short half-life (13.22 h) does not allow for longitudinal studies of pharmacokinetics. ^131^I with a half-life of 8.02 days on the other hand is better suited for long-time physiological studies. This radionuclide, however, contributes to a comparatively higher absorbed dose and a poor image quality, making it less suitable for imaging compared with ^123^I. 

Clinical studies have shown ^124^I to be a quantitative imaging radionuclide for PET-studies of tumor-like objects [2]. A preclinical study with ^124^I labeled antibodies was shown to be quantitative [3]. Furthermore, the long half-life (4.18 days) of ^124^I, allowing a longitudinal study, made it an isotope of choice for thyroid PET Imaging. The sensitivity of PET is higher than that of a SPECT camera allowing for a lower activity to be administered in the thyroid studies. Additionally, the contrast and the spatial resolution of PET images [4] could also exceed that of clinical SPECT images if a positron Range Correction and sophisticated reconstruction algorithms are applied. Furthermore, PET images could suffer less Compton scattering than SPECT imaging due to higher photon energy. Considering these facts, the present study was initiated to investigate if the combined small animal thyroid imaging with the ^124^I PET Imaging could be essential for quantitative thyroid imaging and thyroid dosimetry.

## 2. Material and Methods

### 2.1. Thyroid Phantom

**Figure 1:**
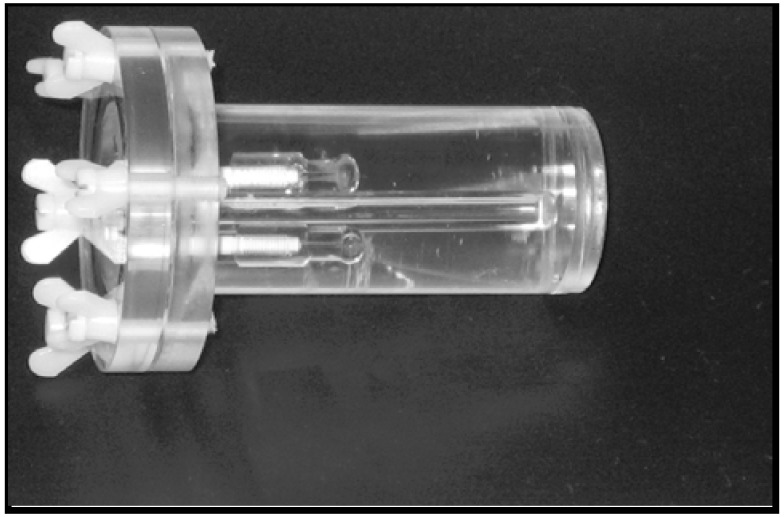
A thyroid phantom with a cylindrical shape (diameter = 30 mm, height = 80 mm) was constructed to imitate the two thyroid lobes on both sides of the tracheal tube.

A cylinder-shaped phantom was used for the validation of the performance of the MicroPET (small animal PET, Siemens Medical Solutions, Inc., USA) and the MicroCAT (small animal Computed Axial Tomography, Siemens Medical Solutions, Inc., USA) imaging systems (Figure 1). The dimension of the cylinder-shaped phantom was 30 mm in diameter and 80 mm in height. Spheres (Data Spectrum Corporation, USA) with inner-volumes of 31 µL, 125 µL and 250 µL, respectively, were placed in the phantom as a set of two spheres with the same size. An air-filled tube, modeling the trachea, was placed in between the two spheres to mimic the rat thyroid. The inner volumes of the spheres were given by the manufacturer. 

The phantom was fully filled with water only (no radioactivity was added to simulate a background activity). The spheres on the other hands were fully filled with a mixture of contrast agent Ultravist^®^ (to enable the CT scan) and different amounts of activity of the ^124^I solution at each PET scan (Table 1). The phantom was then scanned with the same protocols as we usually use for rat scans (see below) in the MicroPET and the MicroCAT scanners. 

**Table 1:** The volumes of the spheres and the activities in the thyroid phantom.

*Volume (µL)*	*Outer diameter (mm)*	*Inner diameter (mm)*	*^124^I-Activity (kBq)*
31	5.95	3.95	140.00
125	8.23	6.23	198.00
250	9.86	7.86	1,765.00

### 2.2. Radionuclide and Animal Preparations

^124^I was purchased from St. Petersburg, Russia (Ainsley Technologies, LLC) and delivered in an activity concentration of 370 MBq in 0.5 mL. Different concentrations of ^124^I activity were diluted with NaCl to a volume of ~0.5 mL before injection into the rats. The activity concentration was measured in a well counter.

Male Wistar (albino) rats, ranging from 354–533 grams, 8–11 weeks age were given different amounts of ^124^I, according to Table 2. The breeding diet included only 1.40 mg/kg iodine. The rats were anesthetized by inhaling Sevofluran (Abbott Scandinavia AB, Sweden) and 1 mL of Hypnorm/Dormicum (5 mg/mL) administered subcutaneously. Thereafter, 0.5 mL of Hypnorm/Dormicum was administered every thirty minutes to keep the rats asleep.

**Table 2:** Rat weights and administered activities of ^124^I.

*Name of Rats*	*Weight [g]*	*Administered* ^124^I-*activity [MBq]*
**Rat 1**	360.00	21.50
**Rat 2**	372.00	18.20
**Rat 3**	354.00	20.70
**Rat 4**	394.00	9.30
**Rat 5**	389.00	5.50
**Rat 6**	533.00	5.40
**Rat 7**	501.00	0.70

The ^124^I activities were injected intravenously, using a neonatal venflon (Neoflon^®^). The residual activity in the syringe and the venflon was measured in the well counter for eventual correction of the rest activity in the syringe.

An activity range between 21.0 and 0.7 MBq was given to the rats. This range was chosen to investigate the injected dose and the thyroid uptake ratio. Rat 7 was given an activity that corresponded to the amount given orally to patients (100 MBq) in thyroid studies scaled to the weight of a rat [5,6,7].

After the experiments, the sedated rats were euthanized. All procedures followed a protocol approved by the ethical committee for use of laboratory animals at the Department of Justice, Denmark.

### 2.3. Imaging Experiments

The 40-min PET thyroid scans, followed by a 10-min transmission scan, using a ^57^Co point source for attenuation correction, were performed. The PET scans were repeated four times at 3, 24, 48 and 72 h for all rats. The PET scans were performed using a small animal PET scanner (MicroPET Focus 120, Siemens Medical Solutions, Inc., USA). The MicroPET system had been cross-calibrated with a well counter to provide a bq/mL unity. The energy window for the emission PET scans was set to 350–650 keV and the time resolution was 6 ns. The energy window for the transmission scans was set to 120–125 keV.

The acquired data for both emission and transmission scans was stored in the list mode and post-processed to obtain 2 bytes 128 × 144 × 32 sinograms. The transmission sinograms were used for attenuation correction of the emission sinograms. Finally, the emission sinograms were reconstructed and resulted in 4 bytes 128 × 128 × 95 image sets with a voxel size of 0.87 × 0.87 × 0.79 mm^3^. The 2D Filtered Back Projection (2DFBP) was used for the image reconstruction process in this work. Furthermore, the emission sinograms were corrected for dead time and decay time. Scatter correction was not applied to the emission data, since the volume of the rat thyroid is small and this issue was assumed to be negligible. The system was calibrated to provide an absolute activity value in a unit of Bq/mL instead of counts per voxel. The final images were then analyzed using the ASIPro Toolbox (Siemens Medical Solutions, Inc., USA).

The small animal CT scans were acquired using the MicroCAT system. A contrast agent Ultravist^®^, 300 mg I/mL (Schering, Germany) (4.5–5 mL) was continuously infused through the Neoflon^®^ throughout the entire scan. The injection of the contrast agent occurred after the injection of the ^124^I activity to avoid any possible interference between the iodine from the contrast agent and ^124^I activity. The acquisition time of each CT scan was 6.5 min generating 360 projections at 360° arc. The X-ray source settings were 70 kVp, 500 μA, and 230 ms for the voltage, the current, and the exposure time, respectively. The CT projections on the 3,000 × 2,970 CCD crystal were binned by four to increase sensitivity and reduce the dataset size. The projections were reconstructed by real-time reconstruction algorithm (the Cobra toolbox) using a Shepp-Logan filter into 768 × 768 × 512 images and voxel size of 0.091× 0.091 × 0.091 mm^3^. The reconstructed CT images were then analyzed using the Amira toolbox (Mercury Computer Systems, Inc., USA).

### 2.4. Activity Measurements

**Figure 2:**
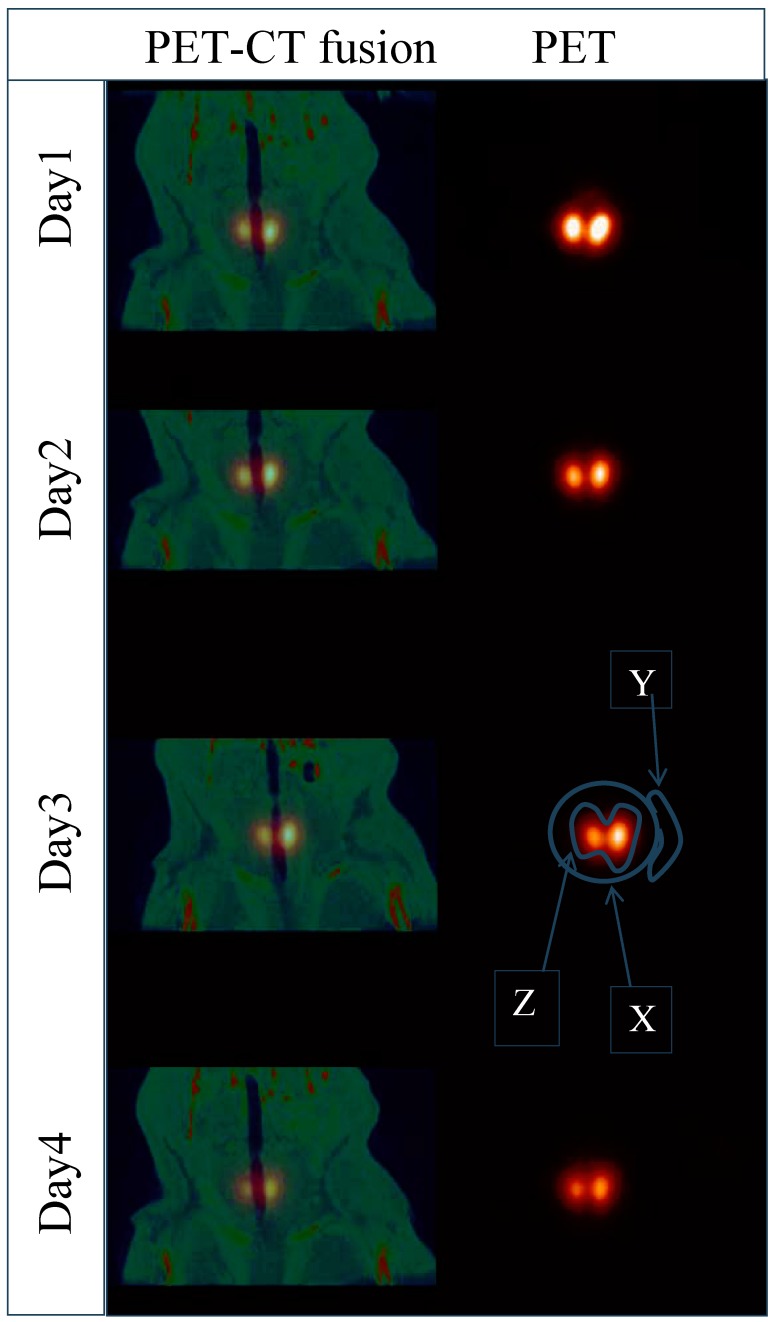
The co-registrated PET/CT and the functional images of one rat in a course of four days are shown. The ROI X is the region of interest that fully covers the thyroid. The ROI Z is the outlining of the thyroid based on CT-images. The ROI Y is a ROI X-neighboring region used for background compensation or spillover activity. The ROI:s are schematic and only for a clarity purposes.

The measured activity in the thyroid in each scan was calculated by outlining the thyroid on the CT images. The outlining of the thyroid was done manually on anatomical images (CT) after an image-co-registration with the corresponding PET images to ensure the exact alignment of the ROI:s on the functional images (PET) (Figure 2). The ROI Z was then applied on the corresponding coronal PET image and the number of pixels that rendered the thyroid was obtained. Even though the CT-based ROI Z could outline the exact shape of the thyroid, counts that might belong to the thyroid region are displaced outside ROI Z due to the limitations of the resolution of the PET modality. To compensate for the count displacement outside the region of interest, a ROI X with a fixed width (20 pixels) in each PET coronal plane that included the thyroid volume was drawn. This was large enough to ensure that the thyroid glands of the rats were completely enclosed. The measured activity in the ROI X is denoted as *A_x_* and the enclosed area is denoted as *n_x_* (No. of pixels). 

ROI X might include counts from the nearby pixels that do not belong to the thyroid. Therefore, a smaller ROI Y (7 pixels) was drawn as close as possible to the ROI X for subtraction of the unwanted counts. The activity *A_y_* in *n_y_* pixels was then calculated.

The total activity of the thyroid is actually the activity in the ROI Z if there is no count displacement in or out of the ROI Z. This assumption could not be true because of the edge blurring due to the limitations in resolution of the PET modality!

For compensation, only the pixels outside the ROI Z were subtracted pixel by pixel with *A_y_*/*n_y_* (the mean activity per pixel in ROI Y). This compensation might correct for the background contribution to the actual thyroid volume and includes the signals displaced outside ROI Z. *A_y_* is the activity measured in an ancillary image background region of *n_y_* pixels. The number of pixels that were compensated for background or spillover activity is *n_x_* – *n_z_*. The number of pixels within ROI Z, *n_z_*, is determined by CT. The thyroid activity (***A****_thyr_* ) can then be expressed as:

                       ***A****_thyr_* = ***A****_x_* – ***A****_y_*/***n****_y_* × (***n****_x_* – ***n****_z_*)                        (1)

### 2.5. Calculation of Mean Absorbed Dose

The mean absorbed dose for the ^124^I in the thyroid was calculated as:


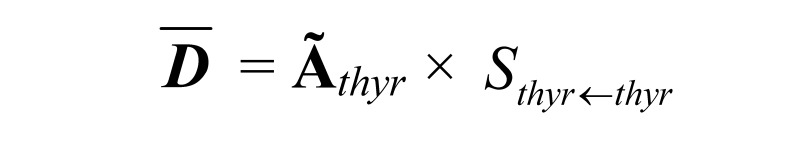
 (2)


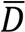
 is the mean *self-absorbed dose* (Gy) in the thyroid. Ã*_thyr_* is the *cumulated activity* (MBqs) in the thyroid. *S_thyr_*_ ← *thyr*_ is the *S-value* (mGy/MBqs). The S-value is specific for the thyroid and was calculated by Monte Carlo simulations with the Electron Gamma Shower code (EGS4) simulated with the MOBY mouse phantom, scaled up by the relative mouse/rat weights. The volumes, determined from CT images, of the thyroid were set individually for each rat with an assumption of a density of 1.04 g/cm^3^ for the thyroid [7,8].

**Figure 3:**
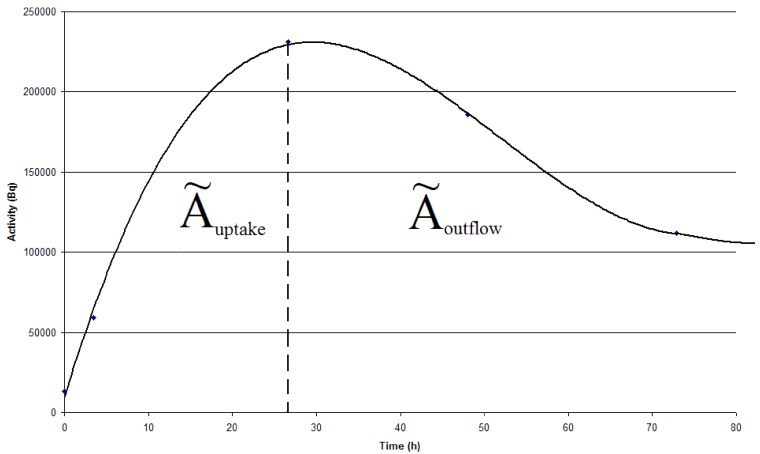
The cumulated activity equals the integral of the time-activity curve, which is a sum of Ã*_uptake_* and Ã*_outflow_* for each animal. Ã*_uptake_* and Ã*_outflow_* are separated by a dotted line.

Ã*_thyr_* was determined individually for each animal by plotting the activity accumulated in the thyroid as a function of time (time activity curve). The integral of the curve equals the cumulated activity and is calculated by defining the curve as a sum of two equations; one for the uptake of the ^124^I activity, Ã*_uptake_* and one for the outflow of ^124^I, Ã*_outflow _*(Figure 3). The Equations can then be used for the calculation of the area under the two integrals. 

### 2.6. Statistics

For comparison of count rates obtained with the well-counting and PET, 95% confidence interval are shown. Non-overlap is taken as a statistical significant difference.

## 3. Results

### 3.1. Performance Evaluation of the MicroPET—Activity Measurements in the Thyroid Phantom

**Figure 4:**
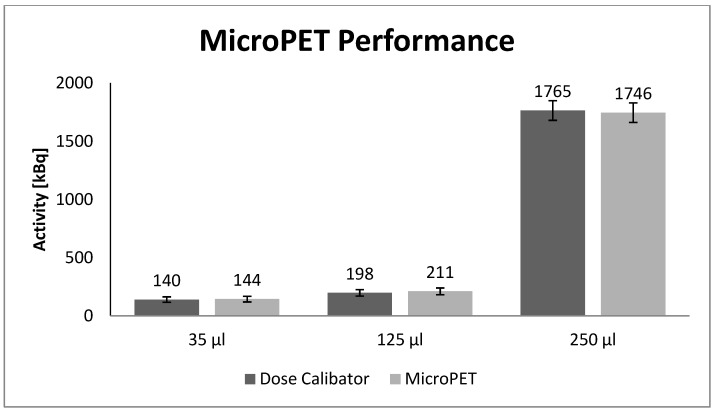
The Comparison between the measured activity in the spheres obtained by the MicroPET and the well counter (dose calibrator), respectively, was done. The measurements of activity in the Spheres with volumes of 31, 125 and 250 μL are shown. The error bars represent the 95% confidence intervals of the Poisson distributed counts.

The results in Figure 4 represent a comparison of the activity in the spheres obtained by an explicit measurement using the well counter and an implicit measurement using the 2D Filtered Back Projection (FBP) reconstructed MicroPET images of the spheres. Since the 95% confidence intervals are overlapping, no significant difference was found between the two methods.

### 3.2. Performance Evaluation of the MicroCAT—Volume Measurements of the Spheres

**Figure 5:**
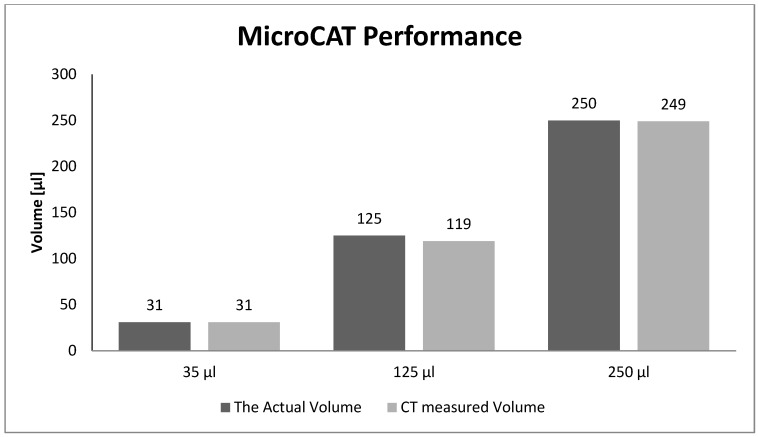
The actual sphere volumes were compared with their corresponding measured volumes from the MicroCAT scans.

The volume measurements of the spheres in the phantom obtained by the MicroCAT scanner were compared with their corresponding actual volumes. The results are represented in Figure 5.

### 3.3. ^124^I distribution in the Thyroid

**Figure 6:**
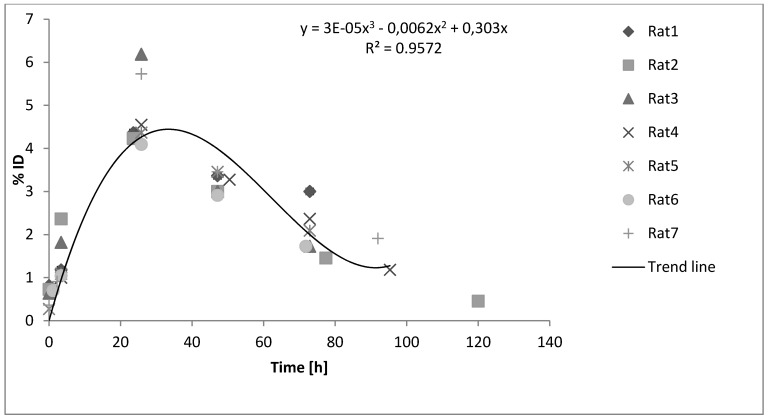
Time-activity curve of ^124^I in the thyroid displaying the biokinetics of iodine in the seven rats. The fraction of the injected activity accumulated in the thyroid during a period of time is shown. The error bars are left out for better visualization of the different points. The trend line is drawn using a least square method in Excel.

The time-activity curves for the seven rats in Figure 6 show the fractional uptake of ^124^I in the thyroids. 

The uptake of ^124^I for all animals showed a similar pattern with a maximum fractional uptake at 24 h post injection between 4.0%–6.2%. 

### 3.4. Absorbed Dose

The S-values used for the absorbed dose calculations were determined by using the thyroid values from our CT images in the MOBY digital phantom (Table 3) [9]. The actual volume of the thyroids (*i.e.*, CT-based volume) could not be used as the input into the Monte Carlo simulation due to rigidity in the simulation program. The Monte Carlo tabulated volumes that were closest to our CT-based volumes were used instead (Table 3).

**Table 3:** S-values and differences between MicroCAT measured volumes and volumes used for Monte Carlo simulations with the EGS4 code and the MOBY mouse phantom.

*Rats*	CT-based Volume (µL)	Monte Carlo Used Volume (µL)	S-value (mGy/MBqs)
Rat 1	49.3	47.3	0.54
Rat 2	41.9	43.4	0.57
Rat 3	47.8	47.3	0.54
Rat 4	70.6	69.8	0.40
Rat 5	45.8	47.3	0.54
Rat 6	34.3	33.2	0.70
Rat 7	51.6	51.3	0.51

## 4. Discussion

The quantification was done on scans with small spheres placed inside the water-filled phantom (Figure 1) with ROI:s large enough to include the annihilation events in the spheres (I-124 positron range of approx. 3.7 mm in water). The activity was enclosed inside the spheres and no activity was added to the surroundings. The reason for not including the background activity was to avoid including any unwanted signals such as signals from the background or Spillover when drawing the ROI:s. The comparison of the activity concentrations in Figure 4 shows a good correlation between the well counter and the MicroPET measurements. 

It is shown in Figure 5 that there were no significant differences between the volume measurements based on CT images and their corresponding true values. 

The visualization of the thyroid using CT technique requires the use of a contrast agent. Since the metabolisms of small animals such as rats is very fast, the retardation of contrast agent in the thyroid is not possible for a long time. We therefore used a continuous injection of the contrast agent throughout the entire scan to avoid the death of the rats and to enable visualization of the thyroid in the CT images. 

Since the thyroids of the rats were healthy, the well-distributed contrast agent contributed to X-ray attenuation in the thyroid that reflected the actual vascular volume. 

Since the CT modality provides reliable *anatomical* information and there is a variation in thyroid volumes of the rats due to their different ages, the CT measured volumes for individual rats were necessary for calculating the S-values as is shown in Table 3. 

The variation in the thyroid volumes of the rats indicates the importance of the CT use for the accurate measurements of thyroid anatomy.

A general observation in Figure 6 is the overall low iodine uptake in the thyroid of rats compared to humans [8,10]. Report No. 5 in the Mird Primer suggests a fractional thyroid uptake of 13.8% after 24 h of oral administrated activity, whereas the result of Johansson *et al*. [10] shows a fractional thyroid uptake of 22%–30% after 24 h. 

Figure 6 reveals that the measuring points tend to spread more after the maximum uptake, indicating a slight difference in biokinetics between the rats. This showed that individual activity measurements are necessary for accurate physiological measurements to determine the absorbed dose into the thyroid. 

It is worth mentioning that the result of Rat 7 in Figure 6, which was injected with a significantly smaller amount of activity compared to the others (Table 1), is well comparable with the result of the rest of the rats. This shows that investigations of the thyroid can be done with as little as 0.7 MBq of ^124^I administered.

Figure 6 shows a higher percentage uptake for Rat 1 than for the rest of the rats after three days. Regarding the spread, the absolute, standard deviation, SD’s are highest at maximum uptake, as there is no larger spread after the maximal uptake. 

The result of the absorbed dose calculations shows that rats 1–3 received an absorbed dose to the thyroid that corresponds to the therapeutically absorbed doses (Table 4) [11]. Rats 4–6 received lower amounts, but it is clear that all of these rats received an absorbed dose that was high. The absorbed dose per unit administered activity was more than 100 times higher than that amount suggested by the Mird Primer for a human thyroid with a maximum thyroid uptake of 5% (38 mGy/MBq).

**Table 4:** Absorbed doses for the seven rats.

*Rats*	Absorbed dose (Gy)	Absorbed dose per unit administered activity (mGy/MBq)
Rat 1	225.7	10,500
Rat 2	82.6	4,500
Rat 3	115.7	5,600
Rat 4	44.7	4,800
Rat 5	38.0	7,000
Rat 6	39.7	7,400
Rat 7	5.2	7,700

## 5. Conclusions

This study shows that quantitative thyroid imaging with ^124^I can be accurately performed with small animal PET combined with small animal CT. The combination of these two modalities can offer a reliable model for longitudinal studies of iodine kinetics that can be translated into the clinic. For absorbed dose calculations, it is essential to determine individual thyroid volumes, which can be performed with contrast infusion and imaging with the MicroCAT. The measurements of the MicroCAT-based volumes showed an inter-individual variation of the size of the thyroid. This confirms the importance of a reliable method to measure the volume of the thyroid individually. This study shows that the combination of small animal PET and small animal CT techniques is essential for quantitative imaging essential for both physiological and therapeutic studies.

## 6. Limitations

The number of rats included in this work is limited; however, it could be sufficient to demonstrate the benefit of using such a model in the small animal thyroid physiology and thyroid dosimetry. The difference in age of the rats could result in a variation of thyroid volumes and different Iodine uptake. The *ex-vivo* measurements of the thyroids of the rats in the well-counter could give valuable information, and future work will include these measurements. 
